# Detection of and response to gender-based violence: a quality improvement project across three secondary mental health services in London

**DOI:** 10.1192/bjb.2024.34

**Published:** 2025-04

**Authors:** Roxanne C. Keynejad, Theo Boardman-Pretty, Sarah Barber, John Tweed, Emily Forshall, Alice Edwards, Joshua Shotton, Claire A. Wilson

**Affiliations:** 1 King's College London, London, UK; 2 South London and Maudsley NHS Foundation Trust, London, UK

**Keywords:** Psychotic disorders/schizophrenia, patients and service users, mental health services, in-patient treatment, community mental health teams

## Abstract

**Aims and method:**

Our team of core and higher psychiatry trainees aimed to improve secondary mental health service detection of and response to gender-based violence (GBV) in South East London. We audited home treatment team (HTT), drug and alcohol (D&A) service and in-patient ward clinical records (*n* = 90) for female and non-binary patients. We implemented brief, cost-neutral staff engagement and education interventions at service, borough and trust levels before re-auditing (*n* = 86), completing a plan–do–study–act cycle.

**Results:**

Documented enquiry about exposure to GBV increased by 30% (HTT), 15% (ward) and 7% (D&A), post-intervention. We identified staff training needs and support for improving GBV care. Up to 56% of records identified psychiatric symptoms related to GBV exposure.

**Clinical implications:**

Moves to make mental healthcare more trauma-informed rely on services first being supportive environments for enquiry, disclosure and response to traumatic stressors. Our collaborative approach across clinical services increased GBV enquiry and documentation. The quality of response is more difficult to measure and requires concerted attention.

Gender-based violence (GBV) refers to acts causing physical, sexual, psychological and other harms perpetrated because of a person's actual or perceived sex, gender, sexual orientation and/or gender identity.^1^ The 2022–2023 Crime Survey for England and Wales found that 27.0% of women and 13.9% of men aged 16 years and over had experienced domestic abuse (the most common form of GBV).^2^ Reported domestic sexual assaults in particular were perpetrated more often against women (9.0%) than men (1.1%). The same data-set found that over 3 years from 2020 to 2022, there were 249 domestic homicides of female victims aged 16 years and older (46.1% of all adult female homicides) and 121 domestic homicides of male victims (8.7% of the total).

GBV is an established social determinant of physical and mental health.^3^ The 2014 Adult Psychiatric Morbidity Survey found elevated adjusted odds ratios (aOR) of past-year suicide attempts (aOR = 2.82), self-harm (aOR = 2.20) and suicidal thoughts (aOR = 1.85) among people who had ever experienced intimate partner violence compared with those who had not.^4^ Adjusted odds of past-year victimisation by any form of violence are around 12 times higher among people with severe mental illness (schizophrenia, schizoaffective disorder, bipolar affective disorder and severe depression) in England than in the general population.^5^ However, trainee psychiatrists often learn more about adverse childhood experiences,^6^ usually without exploration of gender, than traumas sustained in adulthood, such as intimate partner violence, non-partner family-perpetrated violence and (non-domestic) sexual violence.

Despite a clear relationship between violence victimisation and mental ill health, mental health professionals often do not enquire about different forms of current and historical GBV exposure.^7^ Barriers to enquiry include low perceived competence and confidence, and barriers to disclosure include fear of adverse consequences, such as social services involvement, further violence and not being believed.^8^

Trauma survivors advocate a paradigm shift from asking ‘What is wrong with you?’ to ‘What happened to you’?^9^ Such trauma-informed care includes acknowledging links between a broad range of potentially intersecting traumas and mental illness, enquiring sensitively and referring for support, addressing vicarious trauma and re-traumatisation, prioritising trust and amplifying survivors’ strengths.^10^

The high prevalence of GBV and acceptability of routine enquiry among female patients in secondary mental health services in South London are well-established,^11^ irrespective of staff gender,^12^ leading to calls for improved training and referral pathways.^13^ We aimed to improve GBV enquiry and response in secondary mental health services across South East London, using quality improvement methodology.^14^

## Method

### Setting

We conducted this study in services delivered by South London and Maudsley NHS Foundation Trust (SLaM), in the UK. Seeking to capture a range of practice across community and in-patient settings, we focused on three secondary mental health services: a community drug and alcohol (D&A) service, a home treatment team (HTT) and a female in-patient ward.

### Clinical standard

The World Health Organization (WHO) recommends the ‘LIVES’ approach in caring for women subjected to violence: that clinicians (a) listen closely with empathy and no judgement, (b) enquire (‘inquire’) about the person's needs and concerns, (c) validate the person's experiences, showing they believe and understand, (d) enhance the person's safety and (e) support the person to connect with additional services.^15^

### Project design and objectives

Following a plan–do–study–act quality improvement cycle,^14^ our objectives were:

- to determine the proportion of female and non-binary mental health patients with documented enquiry regarding exposure to GBV, currently or in the past
- to intervene to increase the frequency of enquiry, following the ‘LIVES’ framework
- to re-evaluate the proportion of female and non-binary patients with documented enquiry and response to GBV exposure, following intervention.

Our project plans were approved by the SLaM Quality Improvement department. The project was deemed to be service evaluation not requiring ethical approval. This project collected data from medical records only, with the approval of SLaM Audit Office. As this was not a research study and participants were not recruited, informed consent was not obtained.

### Baseline data collection

We extracted anonymised retrospective baseline data from the clinical records of 30 female or non-binary patients from each service's case-load, to determine the frequency of enquiry about exposure to GBV. We also collected baseline data from a community perinatal team and an in-patient mother and baby unit (MBU), which routinely enquire about GBV, as comparators.

Data were entered into an Excel spreadsheet by junior doctors working in the service at the time. Anonymised data were stored securely on password-protected encrypted National Health Service (NHS) trust servers. We recorded the type of abuse and perpetrator relationship (e.g. parent, sibling, partner, friend, stranger). Finally, we noted any temporal links between mental health symptoms or relapses and GBV exposure and whether psychiatric symptoms were related to GBV (e.g. flashbacks, hallucinations or delusions of assault).

### Interventions

**Fig. 1:**
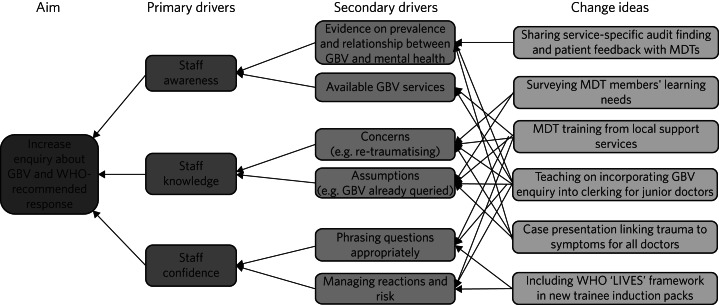
Driver diagram visualising the relationship between our interventions (change ideas), primary and secondary drivers of our overall aim. GBV, gender-based violence; WHO, World Health Organization; MDT, multidisciplinary team; ‘LIVES’, Listen, Inquire, Validate, Enhance safety and Support.

We devised a driver diagram^16^ to visualise hypothesised mechanisms for change within the complex mental health system, based on the literature (Fig. 1). Each change idea focused on practical advice for GBV enquiry and response, to empower staff by increasing primary drivers of GBV awareness, knowledge and confidence. Central to our change ideas was enhancing services’ implementation of WHO's ‘LIVES’ framework for first-line response to GBV.^15^

After baseline audit in March 2022, we implemented change ideas at service, borough and trust-wide levels between May and October 2022. At the service level, each audit lead presented service-specific findings about GBV prevalence to multidisciplinary team (MDT) members. Audit leads also shared with MDT staff the perspectives of female in-patients on an acute ward on how clinical care could be more trauma-informed. These included ensuring that female staff were rostered on every shift, including on emergency teams responsible for restraint (where required), to avoid in-patient experiences triggering and re-traumatising women with a history of GBV. These sessions enabled teams to discuss gaps in enquiry and response, training needs and possible interventions, including ways to facilitate disclosure through the clinical environment (e.g. posters, leaflets, routine enquiry in confidential spaces). In addition to verbal discussions, we electronically surveyed learning needs in relation to GBV among the HTT MDT, as the service model meant that these professionals were less able to attend their team session at the same time.

At the borough level, we delivered teaching sessions to junior doctors (responsible for ‘clerking’ people attending the emergency department or admitted to wards) based at each of the four boroughs served by the trust. The teaching presented definitions and prevalence of GBV and its relationship to mental health, shared the ‘LIVES’ framework, encouraged incorporation into routine assessments and informed learners about local GBV support services. These sessions were in addition to mandatory child and adult safeguarding training and enabled junior doctors to discuss concerns about GBV enquiry and share anonymised experiences of responding to GBV. We also delivered an anonymised clinical case presentation at borough teaching, which enabled discussion of the relationship between trauma and symptoms.

At the NHS trust level, we distributed information about the ‘LIVES’ framework to all new doctors, via induction packs. We organised training for MDT staff of each audited team, delivered by a local GBV support service, which was recorded and made available to those unable to attend. Training addressed GBV, its relationship to mental health, approaches to enquiry and response (including ‘LIVES’), local support services and referral pathways.

### Re-audit

To evaluate whether these interventions had affected enquiry about GBV, we repeated the data collection process for patients randomly selected from the case-loads of the female in-patient ward, HTT and D&A service in November 2022. Owing to high levels of GBV detection by the perinatal team and MBU, we did not re-audit these services.

## Results

Across the three clinical services, we reviewed records of 90 female patients at baseline and 86 at re-audit (a total of 176 unique patient records). We also obtained baseline GBV prevalence data for an additional 59 perinatal service patients (30 for the MBU and 29 treated by the community perinatal team). We therefore audited 235 clinical records in total.

### Demographics

Supplementary Table 1 (available at https://doi.org/10.1192/bjb.2024.34) summarises the age, ethnicity and primary diagnoses of the 149 clinical records audited at baseline and 86 audited post-intervention. All records pertained to females; we identified no non-binary individuals. Median age ranged from 33 years in the HTT (baseline) to 47 years in the D&A service (re-audit).

Across the whole sample (*n* = 235), the largest proportion of clinical records were for patients of White ethnicity (50.3%), followed by Black ethnicity (32.8), Asian (6.9%), mixed (5.1%) and other ethnicities (3.4%); four records (1.7%) did not document ethnicity.

The most common individual primary diagnoses across the full sample were emotionally unstable personality disorder (15.7%), bipolar affective disorder (15.3%), recurrent depressive disorder (14.9%), schizophrenia (11.1%), unspecified non-organic psychosis (10.2%), opioid dependence (7.7%) and schizoaffective disorder (6.8%). Thirty-eight (16.2%) of the overall sample were classified with another primary mental health diagnosis, such as obsessive–compulsive disorder, post-traumatic stress disorder, post-partum psychosis or post-natal depression.

### GBV enquiry, prevalence and symptoms

**Fig. 2:**
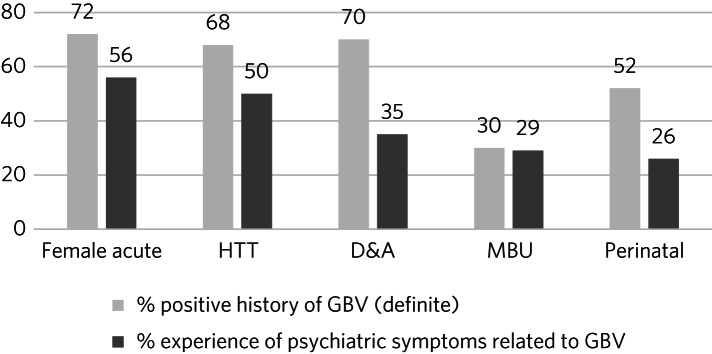
Proportion of clinical records with documented exposure to gender-based violence (GBV) and proportions with psychiatric symptoms related to GBV across five secondary mental health services. Female acute, female psychiatric in-patient ward; HTT, home treatment team; D&A, drug and alcohol service; MBU, mother and baby unit.

Across all records audited (*n* = 235), 26% documented no clear enquiry about exposure to GBV during any episode of care. This varied from 7% in the community perinatal team to 36% on the ward and 38% in the D&A service. Across all clinical records, the estimated overall prevalence of GBV in women with clear enquiry was 62%, with an additional 5% having a suspected (unconfirmed) history of GBV. GBV prevalence ranged from 30% on the MBU to 72% on the ward (Fig. 2).

Intimate partner violence was the most prevalent form of GBV, documented in 77% of records of women reporting any GBV across the five services. Non-partner-perpetrated sexual violence was reported by 36% and non-partner family-perpetrated violence by 15% of all women reporting some form of GBV (categories were not mutually exclusive).

Between 26% (community perinatal team) and 56% (ward) of clinical records included reference to psychiatric symptoms related to GBV (Fig. 2). These included flashbacks of abuse, somatic hallucinations of being assaulted and delusions of assault (in contexts where it was confirmed that no abuse had occurred).

Pre-intervention (*n* = 149), the proportion of participants with enquiry about GBV exposure documented during their current episode of healthcare ranged from 27% on the ward to 50% in the D&A service.

In 77% (182 out of 235) of records audited, enquiry about childhood abuse had been documented. This ranged from 63% in the D&A service to 87% in the HTT. Of those asked, a history of childhood abuse was documented for 45% of patients, and a history was suspected but not confirmed for a further 6%. The prevalence of childhood abuse in those asked (25% across the sample) varied less between services than for GBV.

### Change post-intervention

**Fig. 3:**
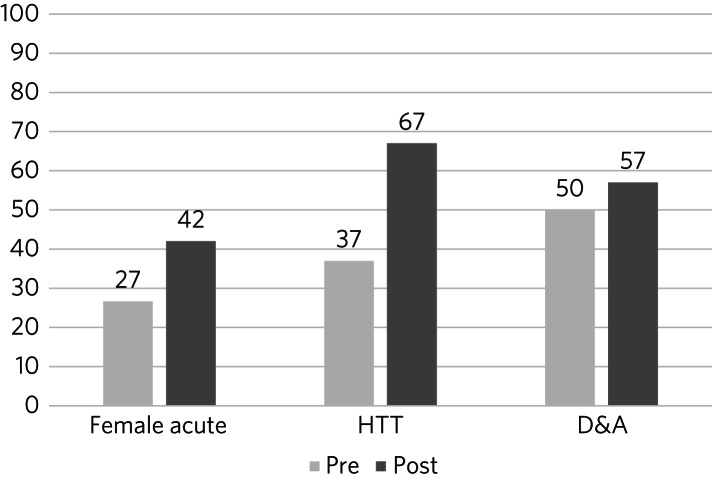
Prevalence of documented enquiry about gender-based violence (GBV) during current episode of care, pre- and post-intervention, across three clinical services. Female acute, female psychiatric in-patient ward; HTT, home treatment team; D&A, drug and alcohol service.

Documentation of GBV enquiry in the random sample of notes reviewed (*n* = 86) increased in each of the three services that we audited pre- and post-intervention (Fig. 3). Enquiry increased by 30% in the HTT, 15% on the ward and 7% in the D&A service.

## Discussion

Evidence from a range of settings supports the acceptability of enquiring about GBV exposure.^12,17^ Although caution has been sounded about potential resource implications, trauma-informed care is likely to improve care quality by making it more person-centred and less re-traumatising.^18^ In our London mental health trust, the overall prevalence of GBV exposure among women with clear enquiry was 62%. The majority of women asked had a history, highlighting the value of routine enquiry and the relevance of GBV to female mental health patients.

### Facilitators of and barriers to enquiry

Moves to make mental healthcare more trauma-informed rely first on services being supportive environments for enquiry, disclosure and response to traumatic stressors such as GBV.^10^ In this quality improvement project, implementing cost-neutral, collaborative interventions at service, borough and NHS trust levels was associated with almost doubling of documented GBV enquiry in some services’ clinical records. The benefits of staff sensitisation to GBV for enhancing enquiry are also supported by high rates of enquiry on the MBU and by perinatal mental health teams, despite neither service using a structured clerking proforma prompting staff to consider GBV.

In secondary mental health services, junior doctors are typically responsible for detailed history taking, where opportunities to routinely enquire about exposure to GBV may arise. Improved GBV detection post-intervention may be attributable in part to the enthusiasm and capacity of junior doctors in our trust to adopt changes to their practice. Research in The Netherlands found that supervisor and peer support of junior doctors was associated with greater work engagement and contributions to quality improvement.^19^ Such factors may have increased engagement with this project in a comparatively well-staffed trust, with few medical trainee vacancies.

However, traumatic and distressing experiences may be disclosed to any member of the MDT, especially where continuity of care is provided by a single professional. Our interventions addressed key learning needs of MDT members regarding GBV, but we noted that shift working, leave, meetings, and clinical and administrative commitments were barriers to integrating GBV enquiry in large teams, beyond medical staff.

Although this project was conducted after the most acute phase of the coronavirus pandemic, lasting impacts of COVID-19 exacerbated burnout, compassion fatigue^20^ and ‘moral injury’.^21^ Lack of protected time and prioritisation are common barriers to staff engagement with quality improvement, whereas leadership support, internal champions and use of data are facilitators.^22^ Therefore, to mainstream a trauma-informed approach across the trust, collaborative leadership with MDT champions and offering support for clinicians’ own histories of trauma are likely to be vital.^23^

### Relationships between GBV and psychopathology

We identified unexpectedly high prevalence of GBV-related psychiatric symptoms among female patients with a history of GBV. Sensitising multidisciplinary professionals to trauma in adulthood, in addition to childhood abuse, may facilitate more trauma-informed explorations of distress arising from psychotic symptoms. Recent studies have demonstrated direct or thematic relationships between the content of hallucinations and childhood trauma.^24,25^ The causation of GBV-related symptoms will be highly complex. However, research exploring thematic links between some psychopathology and GBV in adulthood could inform the development of trauma-informed interventions for survivors.

### Strengths, limitations and implications

Strengths of our study included collaboration between trainee psychiatrists across three diverse services, engagement with local GBV voluntary sector partners and incorporation of patient perspectives into MDT training. Limitations included the lack of non-medical co-leadership and lack of attention at the team level to staff members’ own, including vicarious, trauma,^26^ which is a key feature of trauma-informed care.^9^

Although we were able to measure change in documentation of GBV enquiry post-intervention, capturing the quality of response to GBV disclosures was beyond the scope of this project. Relevant research could apply natural language processing via Clinical Record Interactive Search (CRIS) methods^27^ or use qualitative interviews to explore the quality of response to GBV disclosures in depth and identify focuses for improvement.

Future work should explore ways of formalising referral pathways from mental health to GBV services, engage non-medical GBV champions in each team, establish protocols for responding to GBV disclosure in specific services and consider intersectional factors, such as the additional needs of minoritised groups experiencing GBV. To mainstream a trauma-informed approach at a trust-wide level, collaborative leadership from across the MDT, input from survivors with lived experience and attention to the well-being and trauma histories of staff will be vital.

## Supporting information

Keynejad et al. supplementary material Keynejad et al. supplementary material

## Data Availability

The data that support the findings of this study are available on request from the corresponding author, R.C.K. The data are not publicly available as they containing information that could potentially compromise the privacy of participants.

## About the authors

**Roxanne C. Keynejad** is a National Institute for Health and Care Research (NIHR) clinical lecturer in the Health Service and Population Research department at King's College London, UK and a higher trainee in general adult psychiatry with South London and Maudsley NHS Foundation Trust, London, UK. **Theo Boardman-Pretty** is a higher trainee in general and older adult psychiatry with South London and Maudsley NHS Foundation Trust, London, UK. **Sarah Barber** is an NIHR Academic Clinical Fellow in the Centre for Global Mental Health and Centre for Implementation Science, King's College London, UK and a core psychiatry trainee with South London and Maudsley NHS Foundation Trust, London, UK. **John Tweed** is a higher trainee in general adult psychiatry with South London and Maudsley NHS Foundation Trust, London, UK. **Emily Forshall** is a core psychiatry trainee with South London and Maudsley NHS Foundation Trust, London, UK. **Alice Edwards** is a core psychiatry trainee with South London and Maudsley NHS Foundation Trust, London, UK. **Joshua Shotton** is a core psychiatry trainee with South London and Maudsley NHS Foundation Trust, London, UK. **Claire A. Wilson** is an NIHR Advanced Fellow in the Section of Women's Mental Health at King's College London, UK and an honorary consultant psychiatrist with South London and Maudsley NHS Foundation Trust, London, UK.
