# Supplementary material for: Detection of and response to gender-based violence: a quality improvement project across three secondary mental health services in London
Source: BJPsych Bull. 2024 Apr 19;49(2):93–8. doi: 10.1192/bjb.2024.34 (PMC12014349; doi:10.1192/bjb.2024.34)
Supplement: Keynejad et al. supplementary material [file S2056469424000342sup001.docx]

**Supplementary Table 1: Demographic information**

|  |  | **Baseline audit** | | | | | | | **Post-intervention audit** | | | | | **Total sample** | |
| --- | --- | --- | --- | --- | --- | --- | --- | --- | --- | --- | --- | --- | --- | --- | --- |
|  |  | **Ward** | **HTT** | **D&A** | **MBU** | **Perinatal** | **Total** | **%** | **Ward** | **HTT** | **D&A** | **Total** | **%** | **Total N** | **%** |
|  | | | | | | | | | | | | | | | |
| Age (years) | Median (IQR) | 44 (18) | 33 (22) | 40 (16) | 33 (7) | 34 (7) |  |  | 42 (23) | 35 (18) | 47 (14) |  |  |  |  |
|  | | | | | | | | | | | | | | | |
| Ethnicity: N | White British | 7 | 10 | 14 | 9 | 9 | 49 | 32.9 | 8 | 13 | 22 | 43 | 50 | 92 | 39.2 |
|  | White other | 3 | 2 | 7 | 4 | 6 | 22 | 14.8 | 2 | 1 | 1 | 4 | 4.7 | 26 | 11.1 |
|  | Mixed ethnicity - white and Black | 3 | 1 | 1 | 1 | 1 | 7 | 4.7 |  |  | 2 | 2 | 2.3 | 9 | 3.8 |
|  | Mixed ethnicity – other |  |  | 1 |  | 2 | 3 | 2.01 |  |  |  | 0 | 0 | 3 | 1.3 |
|  | Asian – Chinese |  | 2 |  | 2 |  | 4 | 2.68 | 1 | 1 |  | 2 | 2.3 | 6 | 2.6 |
|  | Asian – Other | 1 | 1 | 2 | 3 | 2 | 9 | 6.04 |  |  | 1 | 1 | 1.2 | 10 | 4.3 |
|  | Black – African | 9 | 3 | 4 | 6 | 1 | 23 | 15.4 | 4 | 4 | 1 | 9 | 10 | 32 | 13.6 |
|  | Black – Caribbean | 1 | 2 |  | 1 |  | 4 | 2.68 |  | 4 | 3 | 7 | 8.1 | 11 | 4.7 |
|  | Black – Other | 6 | 7 |  | 3 | 3 | 19 | 12.8 | 10 | 5 |  | 15 | 17 | 34 | 14.5 |
|  | Other ethnic group |  | 1 | 1 | 1 | 2 | 5 | 3.36 | 1 | 2 |  | 3 | 3.5 | 8 | 3.4 |
|  | Not recorded |  | 1 |  |  | 3 | 4 | 2.68 |  |  |  |  | 0 | 4 | 1.7 |
|  | TOTAL |  |  |  |  |  | 149 | 100 |  |  |  | 86 | 100 | 235 | 100 |
|  | | | | | | | | | | | | | | | |
| Primary diagnosis | Schizophrenia | 10 | 3 |  | 1 | 1 | 15 | 10.1 | 7 | 4 |  | 11 | 13 | 26 | 11.1 |
|  | Schizoaffective disorder | 5 | 1 | 2 | 4 |  | 12 | 8.05 | 4 |  |  | 4 | 4.7 | 16 | 6.8 |
|  | Unspecified nonorganic psychosis |  | 8 |  | 3 | 1 | 12 | 8.05 | 6 | 5 | 1 | 12 | 14 | 24 | 10.2 |
|  | Bipolar affective disorder | 8 | 3 | 1 | 9 | 2 | 23 | 15.4 | 3 | 8 | 2 | 13 | 15 | 36 | 15.3 |
|  | Recurrent depressive disorder | 1 | 7 | 5 | 4 | 9 | 26 | 17.4 | 2 | 4 | 3 | 9 | 10 | 35 | 14.9 |
|  | Emotionally unstable personality disorder | 5 | 2 | 11 | 0 | 3 | 21 | 14.1 | 3 | 5 | 8 | 16 | 19 | 37 | 15.7 |
|  | Other primary mental health diagnosis | 1 | 6 | 2 | 9 | 13 | 31 | 20.8 | 1 | 4 | 2 | 7 | 8.1 | 38 | 16.2 |
|  | Opioid dependence |  |  | 7 |  |  | 7 | 4.7 |  |  | 11 | 11 | 13 | 18 | 7.7 |
|  | Alcohol dependence |  |  | 1 |  |  | 1 | 0.67 |  |  | 3 | 3 | 3.5 | 4 | 1.7 |
|  | Substance use or dependence - other |  |  | 1 |  |  | 1 | 0.67 |  |  |  | 0 | 0 | 1 | 0.4 |
|  | TOTAL |  |  |  |  |  | 149 | 100 |  |  |  | 86 | 100 | 235 | 100 |

D&A: drug and alcohol service, HTT: home treatment team, IQR: inter-quartile range, MBU: mother and baby unit
